# Artificial intelligence inspired design of non-isothermal aging for γ–γ′ two-phase, Ni–Al alloys

**DOI:** 10.1038/s41598-023-39589-2

**Published:** 2023-08-04

**Authors:** Vickey Nandal, Sae Dieb, Dmitry S. Bulgarevich, Toshio Osada, Toshiyuki Koyama, Satoshi Minamoto, Masahiko Demura

**Affiliations:** 1 National Institute for Materials Science (NIMS), Tsukuba, Japan; 2 Department of Materials Design Innovation Engineering, Nagoya University, Nagoya, Japan

**Keywords:** Computational methods, Metals and alloys

## Abstract

In this paper, a state-of-the-art Artificial Intelligence (AI) technique is used for a precipitation hardening of Ni-based alloy to predict more flexible non-isothermal aging (NIA) and to examine the possible routes for the enhancement in strength that may be practically achieved. Additionally, AI is used to integrate with Materials Integration by Network Technology, which is a computational workflow utilized to model the microstructure evolution and evaluate the 0.2% proof stress for isothermal aging and NIA. As a result, it is possible to find enhanced 0.2% proof stress for NIA for a fixed time of 10 min compared to the isothermal aging benchmark. The entire search space for aging scheduling was ~ 3 billion. Out of 1620 NIA schedules, we succeeded in designing the 110 NIA schedules that outperformed the isothermal aging benchmark. Interestingly, it is found that early-stage high-temperature aging for a shorter time increases the γ′ precipitate size up to the critical size and later aging at lower temperature increases the γ′ fraction with no anomalous change in γ′ size. Therefore, employing this essence from AI, we designed an optimum aging route in which we attained an outperformed 0.2% proof stress to AI-designed NIA routes.

## Introduction

Ni-based alloys have been widely used in high-temperature applications such as aircraft industries and gas turbines for several decades due to their exceptional high-temperature strength, excellent resistance to corrosion and oxidation, good ductility, and toughness^1–4^. Though the first “superalloys” started to appear in the 1940s and modern superalloys were developed in the 1980s^4,5^. The optimized chemistries for specific properties of alloy design^6^, metalworking, and heat treatment formulations^7–11^ are still hot topics in the research community. The influence of heat treatment at different temperatures on mechanical properties has been the focus of extensive research and is well documented^12–16^. Much effort has been expended in attempts to manipulate the aging treatment (also known as age hardening or precipitation hardening), which is a critical step to achieve improved performance in the superalloys^13,14,17,18^.

Naturally, even with the same chemical composition of the material and pre-processing history, the material’s properties could differ drastically after selecting different types of aging routes. The optimum combination of microstructural features that result in improved strength in superalloys can be achieved by subjecting them to specific conditions during the solution heat treatment and aging treatment^19,20^. It is crucial to select the suitable temperature for aging treatment in high-temperature alloys^1,2^. Optimum aging treatment is a part of the manufacturing formula for successful commercial products such as, for example, high-temperature Ni-based superalloys with excellent tensile strength and creep properties for land-based and aircraft turbine parts^1,21^. With the development of new alloys, the manufacturer should also need to find the optimal aging scheduling for improved properties. There is a high demand for methodologies that can effectively optimize the aging schedule for these alloys.

For instance, the mechanical properties of Ni-based superalloys are intrinsically governed by the interplay between ordered γ′-L1_2_ Ni_3_ (Al, Ti, Ta) intermetallic compound precipitates with the different morphologies (i.e., cubes, rounded cubes, spheres, or platelets) and disordered γ-fcc Ni solid solution matrix with Co, Mo, Cr, Ta, Hf, Nb, and W elements. In addition, the precipitation hardening by the γ′ precipitates and solid solution hardening significantly improves the mechanical properties in high-temperature alloys^1,15,16,20,22^. The morphology and volume fraction of γ′-precipitate revealed by the aging treatment history and critically affect the mechanical properties of the alloys by acting as barriers to dislocation motions^19,23–28^. Wu et al. found that the precipitates can change from small, spherical particles to larger, elongated particles as aging time increases^29^. This can occur as a result of coarsening, where the particles grow in size due to the diffusion of atoms to the particle surface. In other cases, the morphology of precipitates can remain relatively constant over a range of aging times, particularly if the alloy system is designed to have a stable precipitate morphology, a high density of fine, uniformly distributed precipitates^1^.

The industrial aging treatments could be further improved, which is reflected by rich literature and industrial interest in this subject^1,21^. Latterly, an innovative approach of non-isothermal aging (NIA) has been developed to enhance the mechanical properties of several alloys^30–36^. For instance, there are a couple of examples of alloy systems, such as Fe–Cu^31^, Al–Zn–Mg^32^, Al–Zn–Mg–Cu^12,33,34^, Al–Cu–Mg–Si^35^, and 2A14 Al^36^, which showed the advantage of NIA treatments in achieving enhanced mechanical strength. The influence of NIA investigations on precipitation hardening and microstructure evolution has been studied^31,32,34^ and has achieved excellent results, as summarized in Table 1. For instance, Hutchinson et al.^31^ observed an enhancement in the strength of NIA treatments by 8% compared to the isothermal aging case in Fe–Cu binary alloy. Nicolas et al.^32^ proposed the NIA schedules in which controlling the heating and cooling rate allows modification of the supersaturation matrix and the precipitate critical size, directly impacting the mechanical strength of Al–Zn–Mg alloy. Recently, Zhan et al.^35^ have reported that NIA treatments could enhance the strength of Al–Cu–Mg–Si alloy. It is found that the NIA treatment increases the number density of finer intra-grain precipitates. On the other hand, Huang et al.^36^ investigated the role of NIA treatment in 2A14 aluminum alloys and reported that at the cooling stage of the NIA process, the coarsening of precipitates and the occurrence of secondary precipitation occur concurrently, leading to the attainment of high strength and favorable toughness.

**Table 1:** The NIA cases are discussed in the literature.

Materials	Methods	Remarks	References
Fe–2Cu	Experimental and modeling	Yield strength of NIA improved by 8% more than isothermal aging	^31^
Al–Zn–Mg	Experimental and modeling	NIA increases the precipitate phase fraction	^32^
Al 7050	Experimental	Yield strength of NIA is ~ 5% more than isothermal aging, shortened aging time	^34^
Al–Cu–Mg–Si	Experimental	Yield strength of NIA enhanced by 5.8% than isothermal aging	^35^
2A14 Al	Experimental	Toughness and elongation of NIA increased by 4.4% and 6.5%, respectively	^36^

Furthermore, Jiang et al.^34^ investigated the Al 7050 alloy with varying aging conditions and found the NIA route with a higher yield strength of ~ 5% and shortened aging time than the isothermal aging condition. These observations in the literature show that the number of possible experiments/tests is limited to experimentally determining the optimal time and aging temperatures for these alloys. Another critical reason is that it has been exceedingly challenging to investigate a large number of different types of aging treatment scheduling. Obviously, such optimization is time-consuming and costly with traditional methods due to aging treatment multidimensionality^1,12,37,38^. As a result, it is challenging to determine whether the best solution has been found experimentally.

In the literature, there is no systematic report on NIA scheduling in Ni-based alloys. In addition, we didn’t find suitable methodologies in Ni-based superalloys or another alloy system. From a practical point of view, it could be combined with other statistical modules or tools to eliminate the manual search for optimized cost/performance solutions. We should establish a way to design the NIA scheduling in a huge design-searching space of aging treatment conditions such as aging temperature and aging time.

In this paper, we would like to introduce AI-inspiring methodologies and report our attempt to address this problem for a Ni–Al binary alloy (i.e., Ni-19.11 at. % Al) with the γ/γ′ two-phase microstructure, which is a model for the Ni-based superalloys^39^. Recently, we have developed the computational workflow for high throughput prediction of 0.2% proof stress with different aging treatment scheduling^40^, which is implemented in our original material design system, MInt^41,42^. Herein, we are trying to design NIA schedules with supreme 0.2% proof stress to that obtained by isothermal aging treatment by connecting the prediction workflow with the AI algorithm for efficient searching of NIA. We specifically utilize Monte Carlo tree search (MCTS)^43^, which is a data-driven iterative design algorithm that has demonstrated efficiency in several materials inverse design problems^44^. Taking advantage of the use of computerized prediction methods^45–53^, we deeply analyze the microstructure evolution associated with NIA and examine the essence of AI-found NIA schedules based on expertise in materials science. Based on the specified essence, we finally propose a new concept of NIA design to optimize the high-temperature strength for the Ni-base two-phase alloys, which concept can be called AI-inspired one.

## Computational methods

### Setup for the searching conditions

In this section, we discussed the setup for the searching space conditions. For example, we set the aging time of the total aging scheduling as 10 min. In other words, we would like to design the NIA scheduling, which outperformed the isothermal aging benchmark for 10 min. It is noteworthy to mention that the aging time of 10 min is enough to obtain a reasonable size and volume fraction of γ′ at mild temperatures as the diffusion kinetics in the binary alloys is relatively higher than in the more conventional complex alloy system such as Ni-based superalloys. It should be noted that the NIA is designed for a total fixed time of 10 min with a time frame of 1 min each (i.e., a total number of 10 microstructures for each case, one microstructure for each minute). For example, 700 °C for 1st min, 550 °C for 2nd min, 575 °C for 3rd min … up to 10 min. Therefore, the fixed time of 10 min is considered for comparing the 0.2% proof stress with isothermal aging and NIA schedules.

Here, the digitization parameters employed are described in detail. The entire search space for digitalized conditions is ${9}^{10}$ (i.e., 3,486,784,401). For instance, the considered temperature range for scheduling is 500–700 °C with 25 °C intervals for the further optimal solution search for the NIA schedule. As a result, the total considered temperature for scheduling is 9 (for example, 500, 525, 550, 575 °C…700 °C), and the total considered time interval is 10 (i.e., 1, 2, 3 min …10 min). Therefore, the optimized NIA schedules are obtained from a huge search space by the MCTS algorithm.

**Figure 1:**
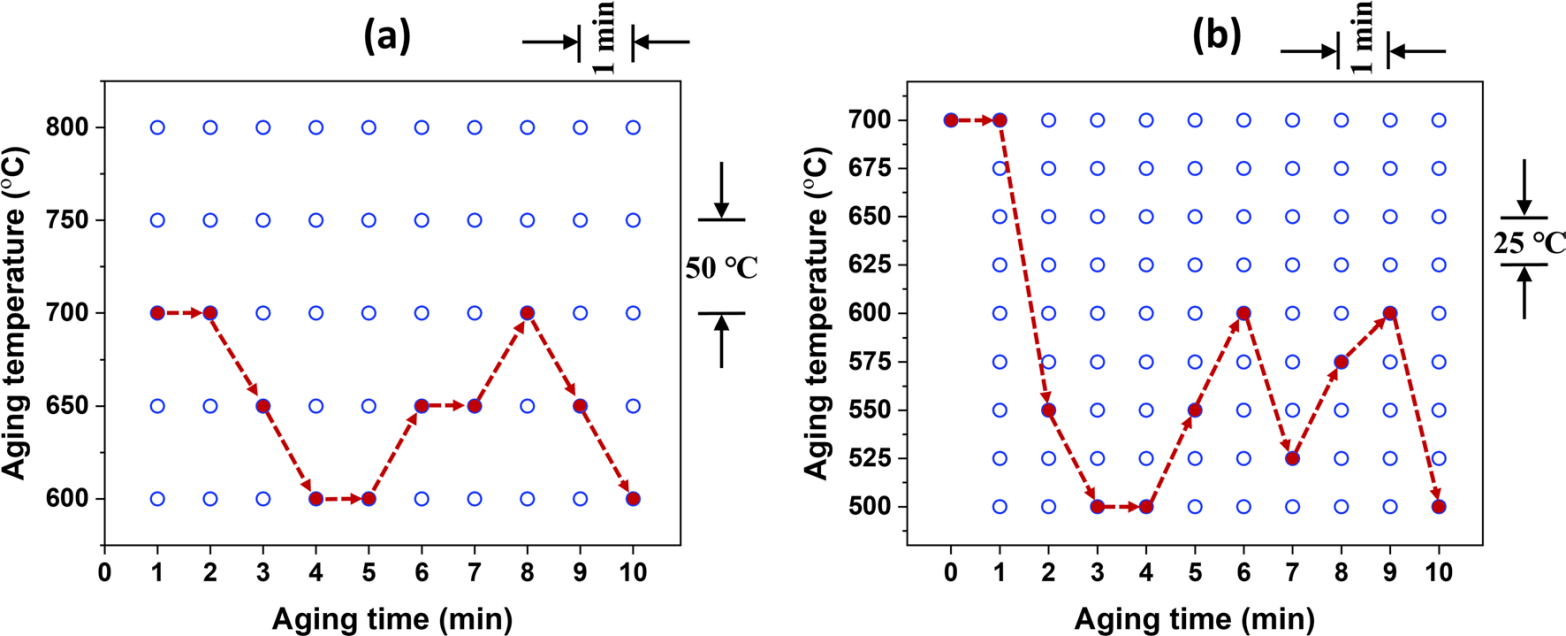
The plots show the digitalized parameters for the (**a**) coarse-tuned and (**b**) fine-tuned MCTS NIA schedules.

Figure 1a illustrates the coarse-tuned digitalized parameters (i.e., temperature interval and time step size) that the MCTS algorithm utilized to choose the starting temperature during the NIA scheduling. For example, the aging temperature input parameter for starting temperature is set to be in the range of 600–800 °C with an interval of 50 °C and for the fixed time of 10 min, as shown in Fig. 1a. After coarse tuning for the starting temperature, the aging temperature range is considered for the fine-tuning to find the optimal solution, as illustrated in Fig. 1b. Finally, in this fine-tuned step, the types of the NIA reached up to ~ 3 billion.

### Monte Carlo tree search

**Figure 2:**
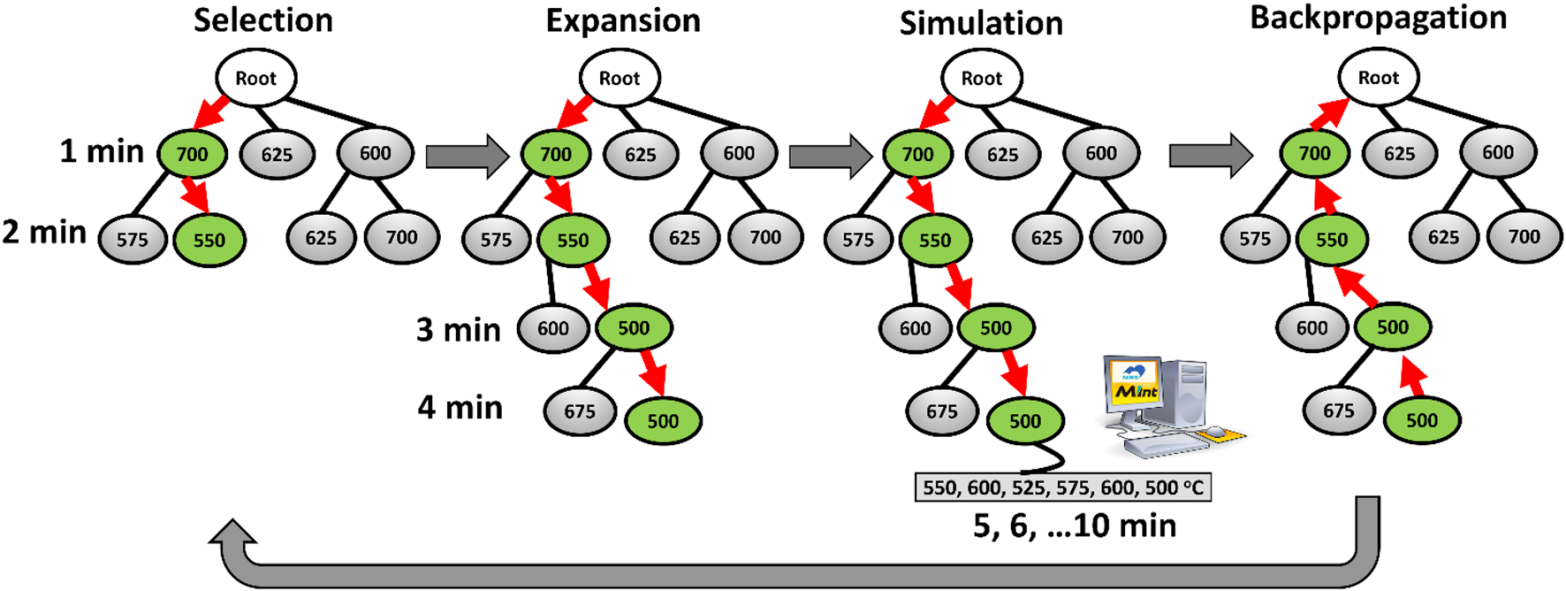
Monte Carlo tree search (MCTS) for a Ni–Al binary alloy system. The different types of NIA space are represented as a shallow tree where each node represents a possible end temperature at a certain step in the NIA route assignment. A route from root to a leaf in the maximum depth represents a full NIA schedule. A full schedule can be obtained from such a tree by using the random rollout technique. The tree is expanded iteratively towards the promising area of the search space. Each iteration consists of 4 steps: selection, expansion, simulation, and backpropagation.

MCTS is an iterative, guided, random best-first tree search algorithm that systematically explores a space of types of NIA schedules to determine the optimal solution to a problem. In this work, we have used a combination of the MInt system and an AI-based search algorithm, MCTS, to design NIA schedules. To design the NIA, a Python implementation of MCTS named MDTS was used as it automatically and adaptively balances the search exploration vs. exploitation hyperparameter^54^. In the case of MCTS design, the different types of NIA schedules are represented using a shallow tree, where each node in the tree is a possible end temperature assignment for a single step in the schedule, as illustrated in Fig. 2. A complete path from the root to a leaf in the maximum depth represents a full NIA schedule. In the beginning, the tree has no nodes. The search starts with random rollout selection, and then the tree expands gradually towards the promising area of the search space using previous observation. The tree is traversed iteratively from the root, following the path with the most promising path using the Upper Confidence Bound score (UCB)^43^. In each iteration, 4 steps are conducted, selection using UCB score, expansion where a new node (possible NIA schedule step is added in the tree), simulation, where the route in the tree is completed using random roll out and the obtained schedule is evaluated using Mint system, finally, in the backpropagation step, tree information is updated for a more informed decision in the next iteration (see Fig. 2). We also computed the case of conventional isothermal heating and specified the best temperature to obtain the highest 0.2% proof stress at a service temperature. It is important to highlight that schedules obtained from the MCTS design are compared with the benchmark obtained from the isothermal aging conditions.

### Simulation system, MInt

**Figure 3:**
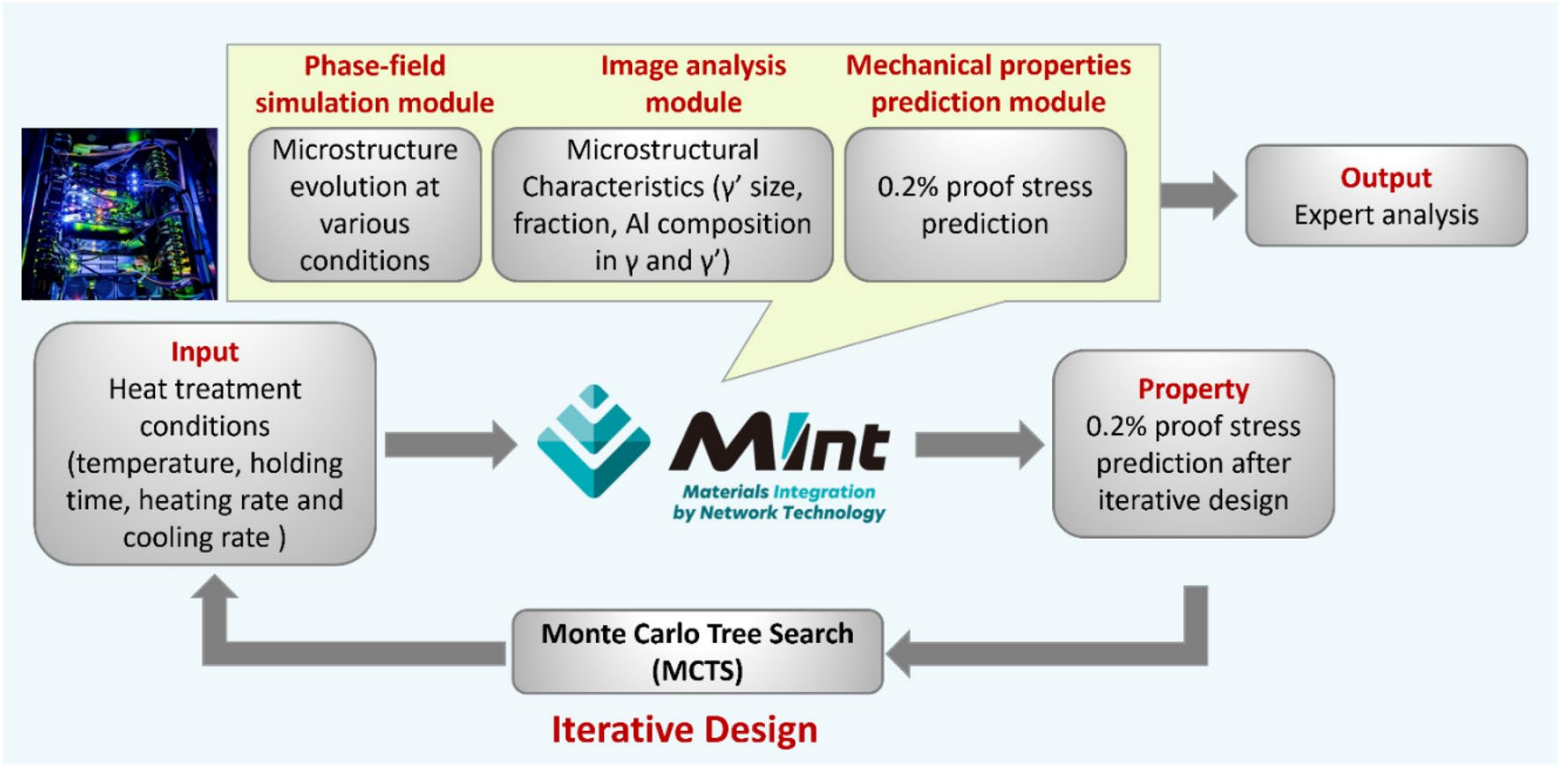
Outline the MCTS-designed NIA routes by computational workflow management.

In this section, we introduce the MInt system, which is used for the forward calculation in these alloys. MInt system is a computational workflow that we developed to simulate the microstructure evolution and evaluate the high-temperature strength for different alloys^41,42^. The detailed architecture of the MInt module system can be referred to in references^40,42^. The module was built as part of the Cross-ministerial Strategic Innovation Promotion Program (SIP) project on materials integration (MI)^41^. It consists of the microstructure prediction at different conditions using phase-field simulation^55^ for simulated microstructure image analysis, which includes γ′ size (the mean diameter of a circle of equal projection area for γ′-precipitate) as well as volume fraction and the performance prediction (i.e., 0.2% proof stress)^19,56^, as schematically shown in Fig. 3.

### Alloy system and initial microstructure

**Figure 4:**
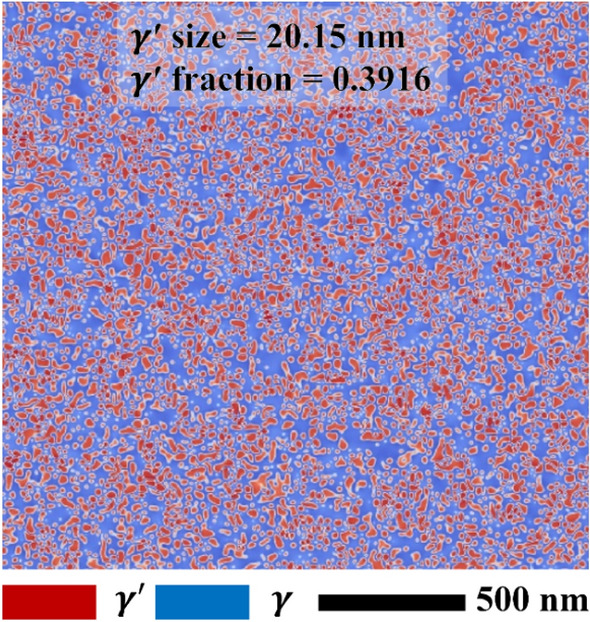
Initial microstructure (prior to NIA scheduling) of Ni-19.11 at. % Al alloy, annealed at 1000 °C for 32 s.

The composition of the modeled alloy is Ni-19.11 at. % Al. In this study, we assumed that there are no heterogeneities (i.e., no dendritic structures) in the starting microstructure. Therefore, the microstructure is in a completely homogenized state. The microstructure was obtained just after the complete homogenized heat treatment. Technically, phase-field modeling cannot treat precipitation. It should be noted that the present phase-field model simulates the evolution of microstructure during growing of the γ′ according to the partial differential equations^57^, including the diffusion and Gibbs free energy. To mimic the assumed initial microstructure, we prepare the initial microstructure with the phase-field simulation after isothermal annealing at 1000 °C for 32 s (i.e., prior to the NIA scheduling), which gives very fine γ′ precipitates in the microstructure with supersaturated γ, as shown in Fig. 4. It reasonably agrees with previously available experimental evidence provided by Osada et al.^40^ for the Ni-19.11 at. % Al alloy (i.e., using a similar alloy composition). The initial microstructure characteristics, such as alloy composition, γ′ precipitate size, and volume fraction of γ′ precipitates, are tabulated in Table 2. For example, the γ′ size and volume fraction is given as 20.15 nm and 0.3916, respectively. The volume fraction of the initial microstructure is far from the equilibrium state. From this, the γ′ grows, and diffusion of Al happens during the aging treatment from the non-equilibrium state.

**Table 2:** Initial microstructure characteristics of Ni-19.11 at. % Al alloy.

Alloy composition	γ′—precipitate size	Volume fraction of γ′
Ni-19.11 at. % Al	20.15 nm	0.3916

## Results and discussion

### Isothermal aging benchmark

**Figure 5:**
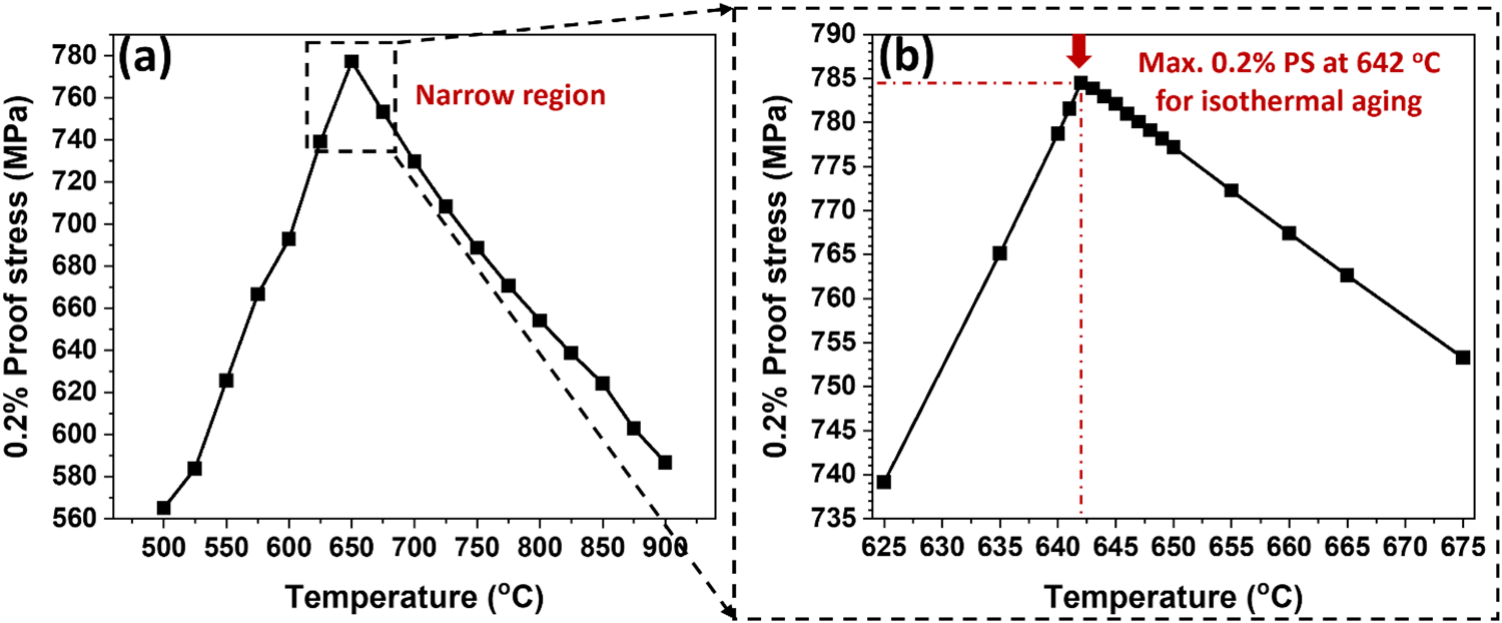
Variation of the (**a**) 0.2% proof stress at 725 °C as a function of aging temperature in the range of 500–900 °C and (**b**) fine-tuned investigation to obtain benchmark 0.2% proof stress in the range of 625–675 °C (*0.2% PS*: 0.2% proof stress).

In this section, the isothermal aging benchmark has been obtained from the calculated 0.2% proof stress of the Ni-19.11 at.% Al alloy with γ–γ′ two-phase as a function of the aging temperature using the MInt system, as shown in Fig. 5. These calculations have been performed for temperatures from 500 to 900 °C with the temperature interval of 25 °C for fixed 10 min. It is apparent that the 0.2% proof stress increases with aging temperature until it reaches its maximum value (i.e., peak-aged state) and then gradually drops, as illustrated in Fig. 5a. The benchmark (i.e., highest 0.2% proof stress) for the isothermal aging condition at a service temperature (i.e., 725 °C) with a fixed time of 10 min was obtained; the best isothermal aging temperature was 642 °C. The obtained benchmark 0.2% proof stress is found to be 784.48 MPa. This condition is further referred to as the isothermal aging benchmark. It should be noted that the obtained benchmark value is examined up to a narrow range of 1 °C, as shown in Fig. 5b.

**Figure 6:**
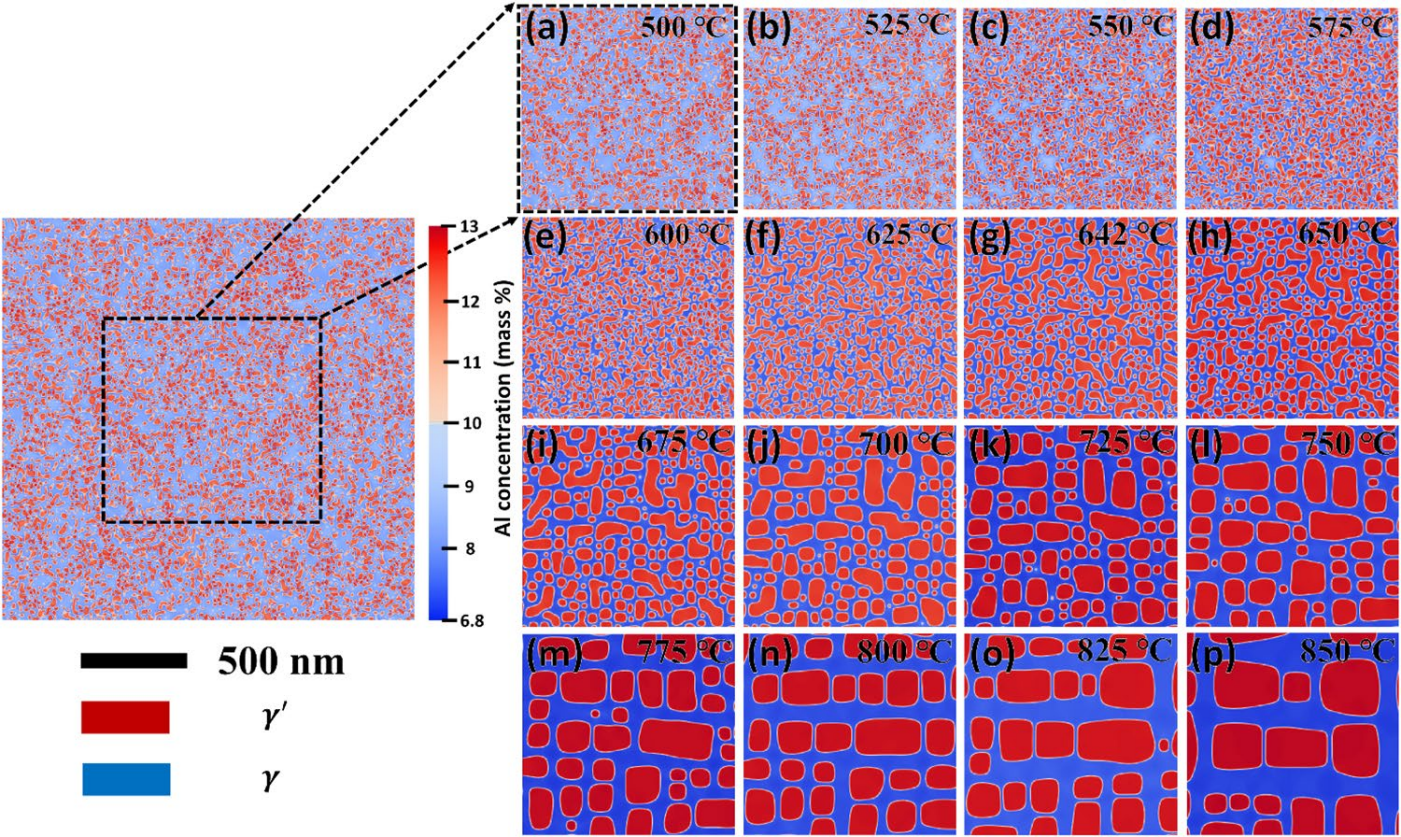
Simulated phase-field microstructure showing the evolution of γ′ precipitates at 10 min in Ni–Al binary alloy at different isothermal aging temperatures of (**a**) 500 °C, (**b**) 525 °C, (**c**) 550 °C, (**d**) 575 °C, (**e**) 600 °C, (**f**) 625 °C, (**g**) 642 °C, (**h**) 650 °C, (**i**) 675 °C, (**j**) 700 °C, (**k**) 725 °C, (**l**) 750 °C, (**m**) 775 °C, (**n**) 800 °C, (**o**) 825 °C, (**p**) 850 °C. The microstructures are all zoomed-in images of simulated $2 \mu \mathrm{m}\times 2\mu \mathrm{m}$ phase-field microstructure, as illustrated, for instance, in (**a**).

It is essential to show the simulated microstructural evolution for these conditions to understand the microstructural features. A clear and distinguishing feature of the microstructural evolution of γ′ precipitates as a function of aging temperature at the fixed aging time of 10 min in Ni–Al binary alloy is illustrated in Fig. 6. One can notice that the aging temperature heavily influences the microstructures. Finally, it is evident from the microstructures that coarsening of γ′ precipitates are significantly promoted after isothermal aging at temperatures more than 650 °C, as highlighted in Fig. 6h–p. In addition, the coarsening kinetics of the γ′ precipitates accelerate (see Fig. 6) as the precipitation hardening slows down as the aging temperature increases to 850 °C, resulting in a considerable (around 200 MPa) decrease in the 0.2% proof stress (refer to Fig. 6a). This may be related to the drop in number density of precipitates during the rapid coarsening (i.e., increase in interparticle spacing), which directly minimizes the obstruction to the dislocations by the precipitates in the system. It is noteworthy to mention that the critical size of γ′ is found to be around 41 nm (Supplementary Fig. S1, see online supplementary material), over which the over-aging occurred. The microstructures in Fig. 6a–p are zoomed-in images of simulated $2 \; \upmu \mathrm{m}\times 2 \; \upmu \mathrm{m}$ phase-field microstructures. It should be noted that the phase-field simulation is performed under periodic boundary conditions. Therefore, no inconsistencies are observed at the edges of microstructures. The detailed quantitative analysis of the microstructure evolution of Fig. 6 (Supplementary Fig. S1) and contour plots (Supplementary Fig. S2) as a function of aging temperature is given in the supplementary material.

### Optimizing the NIA schedules using AI

**Figure 7:**
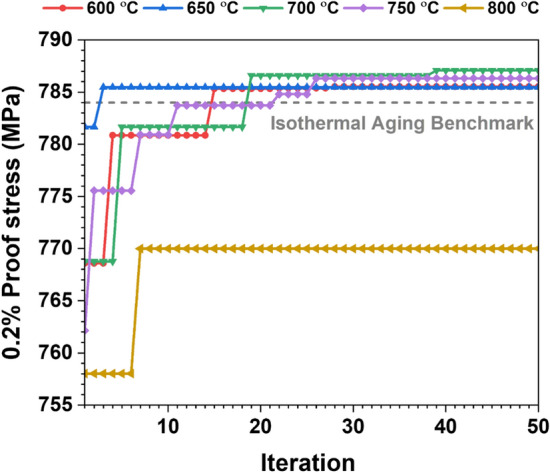
Variation of maximum 0.2% proof stress with the number of iterations as a function of different starting temperatures for a fixed time of 10 min.

In order to promote solid solution hardening and age hardening, the starting temperature plays a vital role. For instance, a higher starting temperature causes γ′ particles to coarsen more rapidly in the early state and with less tendency to nucleation than at lower temperatures^1,39^. However, an optimum starting temperature is required for the pronounced precipitation hardening effects with aging time. At the same time, we adjust the starting temperature range from 600 to 800 °C with an interval of 50 °C between each temperature selection (as discussed in Fig. 1a). Figure 7 shows the variation of maximum 0.2% proof stress with the number of iterations as a function of starting temperature.

It should be mentioned that each data point of the heat-treated sample in the plot corresponds to the NIA condition, which was heat treated for a fixed time of 10 min. Therefore, the relationship between 0.2% proof stress and the number of iterations has been established. The results revealed that a starting temperature of 700 °C (green curve) has superior 0.2% proof stress (after the 20th iterations) to the isothermal aging benchmark, as shown in Fig. 7. In contrast, the 650 °C (blue curve) achieved the 0.2% proof stress value after 2 iterations but it is less than 700 °C. Hence, we selected 700 °C as the starting temperature for fine-tuned searching for further NIA scheduling.

**Figure 8:**
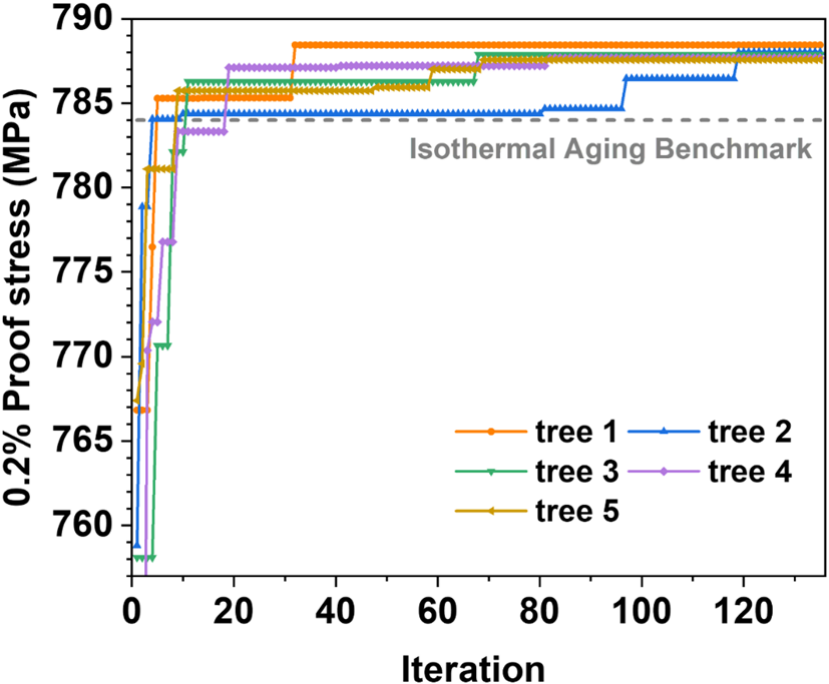
Fine-tuned NIA scheduling for 0.2% proof stress versus the number of iterations at starting temperature of 700 °C for 10 min.

Figure 8 shows the more fine-tuned NIA searching to obtain the optimum 0.2% proof stress at the starting temperature of 700 °C for a fixed time of 10 min by the MCTS design. For instance, the total 12 independent MCTS trees are considered with a larger number of iterations of 135 for each tree as per computational budget. We plotted the iteration curve for the case finding the best five NIA schedules. It is noteworthy to mention that all these five trees have multiple types of NIA, which outperformed the isothermal aging benchmark, as shown in Fig. 8. The plot also demonstrates how the AI explored a huge search space to select the best NIA schedules.

The results also showed that the AI discovered NIA schedules (tree 1, orange curve) in fewer than 5 iterations, outperforming the isothermal aging benchmark (grey dotted curve). In the case of tree 4, it achieves the NIA that outperforms the isothermal aging benchmark after 20 iterations. In this way, most of the trees find the supreme 0.2% proof stress case in the very early stage. In contrast, depending on the trees, sometimes after 50–60 iterations, AI cannot find the NIA schedule that performs better. MCTS discovered substantially better examples as a result of these incubations. Interestingly, we successfully obtained 110 NIA schedules out of 1620 NIA that outperformed the isothermal aging benchmark.

**Figure 9:**
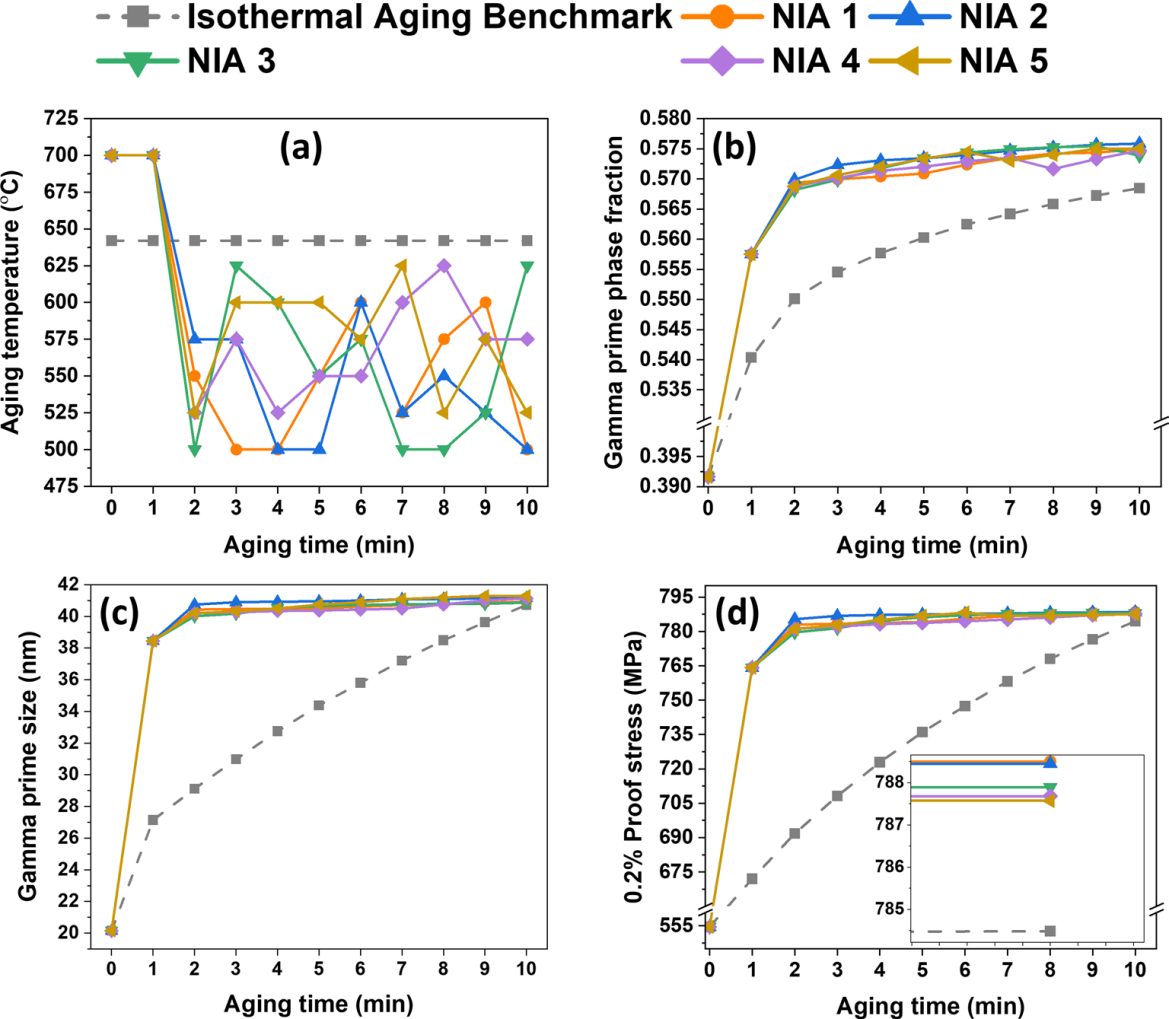
(**a**) Sketch map of NIA scheduling processes in this study, (**b**) γ′ phase fraction, (**c**) γ′ size and (**d**) 0.2% proof stress as a function of aging time (the reader is referred to the web version of this article for interpretation of the color references in this figure legend).

Furthermore, by using the 10 time frames of 1 min each, the MCTS discovered several NIA routes that outperformed the best isothermal aging value, leading to the higher 0.2% proof stress by tuning the combination of heating and cooling rates (for example, cooling from 700 to 550 °C, the cooling rate is $-2.5 \mathrm{^\circ{\rm C} }/\mathrm{s}$) in each time frame, as illustrated in Fig. 9a. Figure 9b–d compare the microstructure characteristics (for example, γ′ phase fraction and size) as well as 0.2% proof stress of best five outperformed NIA by the isothermal aging benchmark. For instance, the precipitation hardening process during isothermal aging and NIA as a function of aging time up to 10 min is demonstrated in Fig. 9d.

For the example of isothermal aging, it can be noticed that the 0.2% proof stress increases steadily as the aging time increases from time 0 to 10 min (see grey dotted line in Fig. 9d). In contrast, when an alloy is subjected to 700 °C for one minute for the case of NIA, the 0.2% proof stress increases significantly within the first 2 min (i.e., 1 min at 700 °C from starting temperature of 700 °C and 1 min during cooling from 700 °C to lower temperatures such as 550 °C), as shown in Fig. 9d. It can be attributed to the increase in γ′ precipitate size to ~ 40 nm within 2 min of aging, as illustrated in Fig. 9c. The phase fraction also rises remarkably during this stage and higher phase fraction of ~ 56% is obtained in this time period. Following that, the size nearly stabilizes, and the fraction increases from 56 to 57.7% (see Fig. 9b).

**Figure 10:**
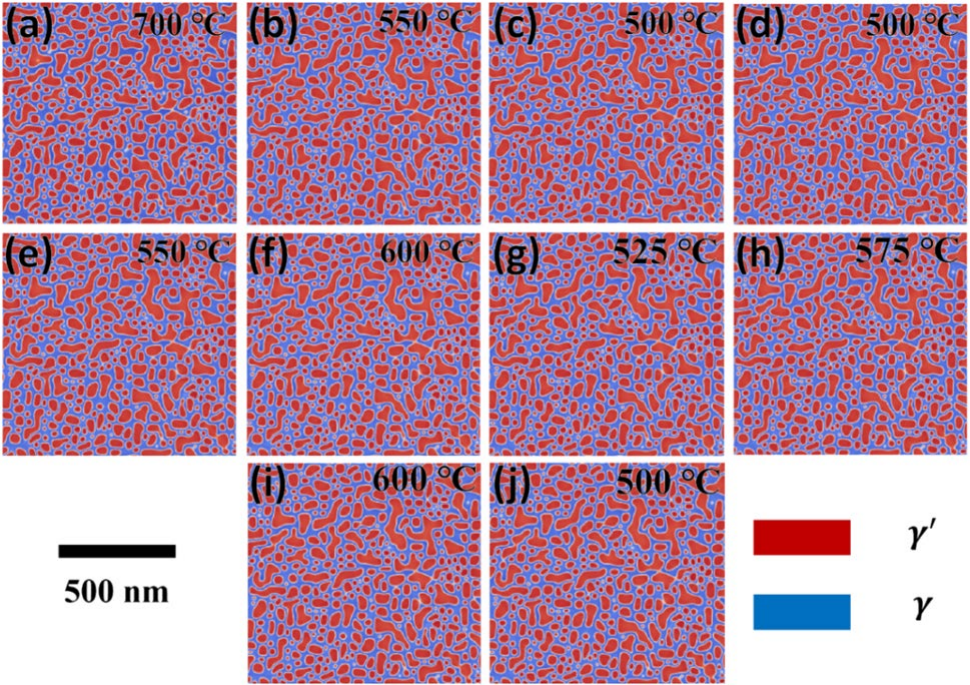
Simulated phase-field microstructure of best performed NIA (i.e., NIA 1) route proposed by MCTS at different stages (interval of 1 min time frame) of scheduling at (**a**) 700 °C, (**b**) 550 °C, (**c**) 500 °C, (**d**) 500 °C, (**e**) 550 °C, (**f**) 600 °C, (**g**) 525 °C, (**h**) 575 °C, (**i**) 600 °C, (**j**) 500 °C.

It is interesting to note that the higher 0.2% proof stress, which is more prominent than the 0.2% proof stress of the isothermal aging benchmark value, is obtained in the case of NIA conditions in just 2 min (refer to Fig. 9d). It is evident that many NIA scheduling theoretically offers a 0.2% proof stress that is higher than the isothermal aging benchmark (see the inset in Fig. 9d). For instance, the microstructural observations of one of the best NIA cases (i.e., having 0.2% proof stress of ~ 789 MPa) clearly illustrate the highly stable nature of γ′ precipitate in simulated phase-field microstructures in these alloys, shown in Fig. 10. The result indicates that the aging temperature is relatively low in the later stages of NIA (see Fig. 9a), and the coarsening kinetics is expected to be weaker, as seen in Fig. 10c–j. At lower temperatures, the precipitate becomes much more stable. Thus, even though the fraction increases, the precipitate size does not vary significantly. However, this is shown to be a favorable factor for increasing strength.

**Figure 11:**
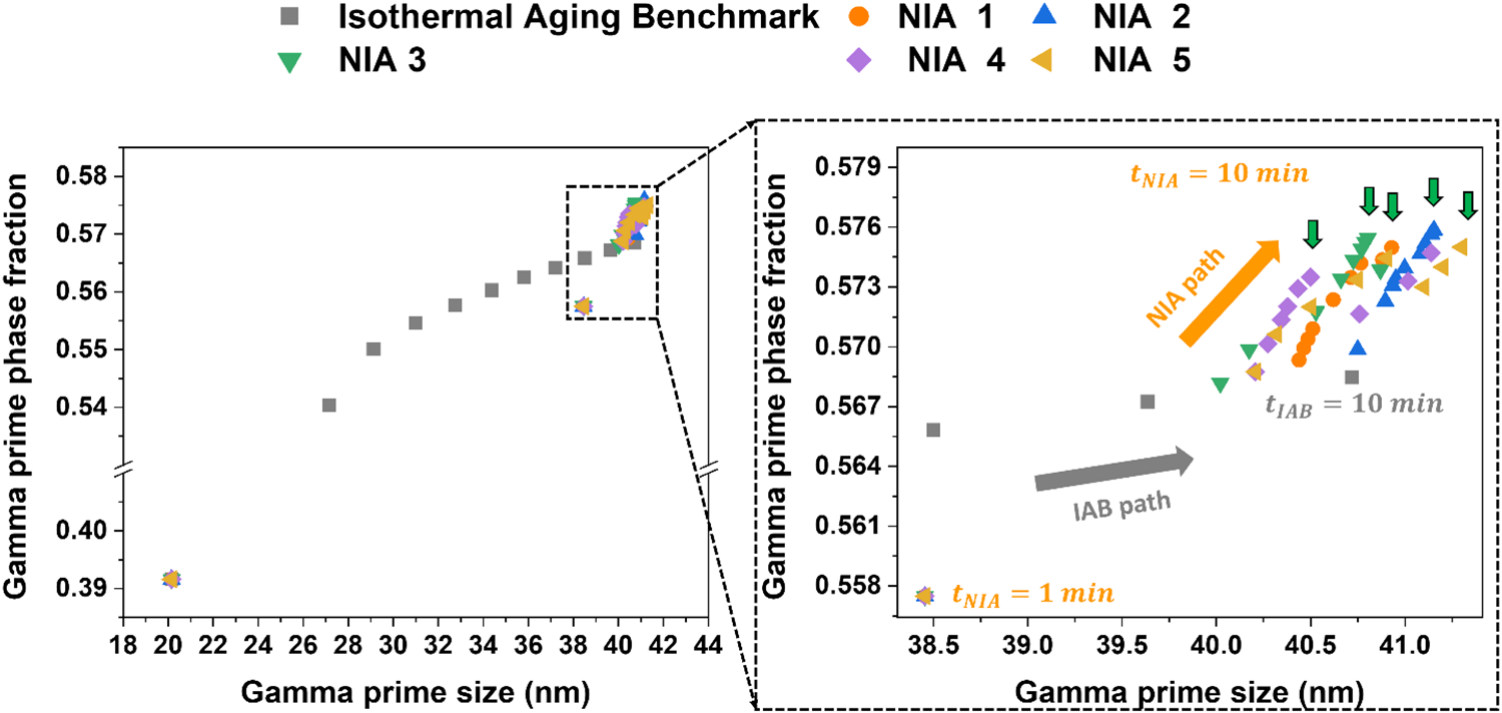
Variation of γ′ phase fraction with γ′ size of isothermal aging benchmark and outperformed NIA schedules. A black dotted box highlights the zoomed-in image (*IAB* isothermal aging benchmark).

A useful way of visualizing the correlations between γ′ phase fraction and γ′ size exhibited by this alloy is plotted in Fig. 11. It clearly shows the statistical and comparative results of the γ′ phase fraction and γ′ size of the top five NIA schedules, which performed better than the isothermal aging benchmark. The plot illustrates the statistical results of the isothermal aging benchmark at 10 min which are similar to those at 2 min for NIA conditions. For instance, the average γ′ precipitate size in NIA (shown by the orange symbol in the zoomed-in Fig. 11) is almost similar to the precipitate size in the isothermal aging benchmark. As the aging time for NIA scheduling increases from 2 min to 10 min, the phase fraction increases, followed by the NIA path, as shown in zoomed-in Fig. 11. The size of γ′ precipitate increases first and then stabilizes as the aging time increases. Therefore, optimum NIA scheduling routes are obtained, which provide a higher phase fraction (~ 57.7%) and critical γ′ precipitate size (~ 41 nm). It is clearly observed that the upward path is followed by the NIA schedules (as indicated by the green arrows in Fig. 11).

The above analysis implies the essence of the NIA, the top 5 NIA schedules discovered by AI have some common features, such as early high-temperature aging for a shorter time, which quickly increases the γ′ precipitate size to near critical size in the early stage and then, followed by the lower temperature aging to increase the γ′ phase fraction by keeping the γ′ under the critical size. The early-stage high-temperature aging (in this case, 700 °C) increases the γ′ size near the critical size (~ 41 nm). It is essential to keep the duration of the high temperature short and immediately lower the temperature. This is because if the size of γ′ increases more than the critical size, the number density (i.e., distribution of precipitates in the microstructure) of γ′ reduces, yielding an over-aging. According to Osada et al.^19^ the number density of the secondary γ′ within the trimodal distributed γ′ (i.e., primary, secondary and tertiary γ′ precipitates) in a Ni-based disk superalloy plays a significant role in precipitation strengthening. Furthermore, to increase the phase fraction of the γ′ precipitates, a subsequent aging at a relatively lower temperature is also effective in enhancing the strength. The γ′ is more stable at lower temperatures^1,39^ and thus, the volume fraction was assumed to increase slightly at the lower-temperature aging.

### AI-inspired expert-designed NIA

Based on the above-mentioned discussion, we can understand that the AI-founded top 5 NIA commonly consists of two steps, as follows: Step 1. High-temperature, short-time aging; and Step 2. Low-temperature, long-time aging. The question arises whether the small and complex temperature changes in Step 2 (see Fig. 9a) are not essentially necessary. In other words, we considered that it was possible to design a much simpler NIA and attain higher 0.2% proof stress by employing the essence of AI. Then, we designed a simple two-step aging consisting of 1-min isothermal aging at 700 °C, cooling down to a lower temperature, and 8-min isothermal aging at the lower temperature. This newly proposed NIA route can be referred to as AI-Inspired expert-designed NIA schedule.

**Figure 12:**
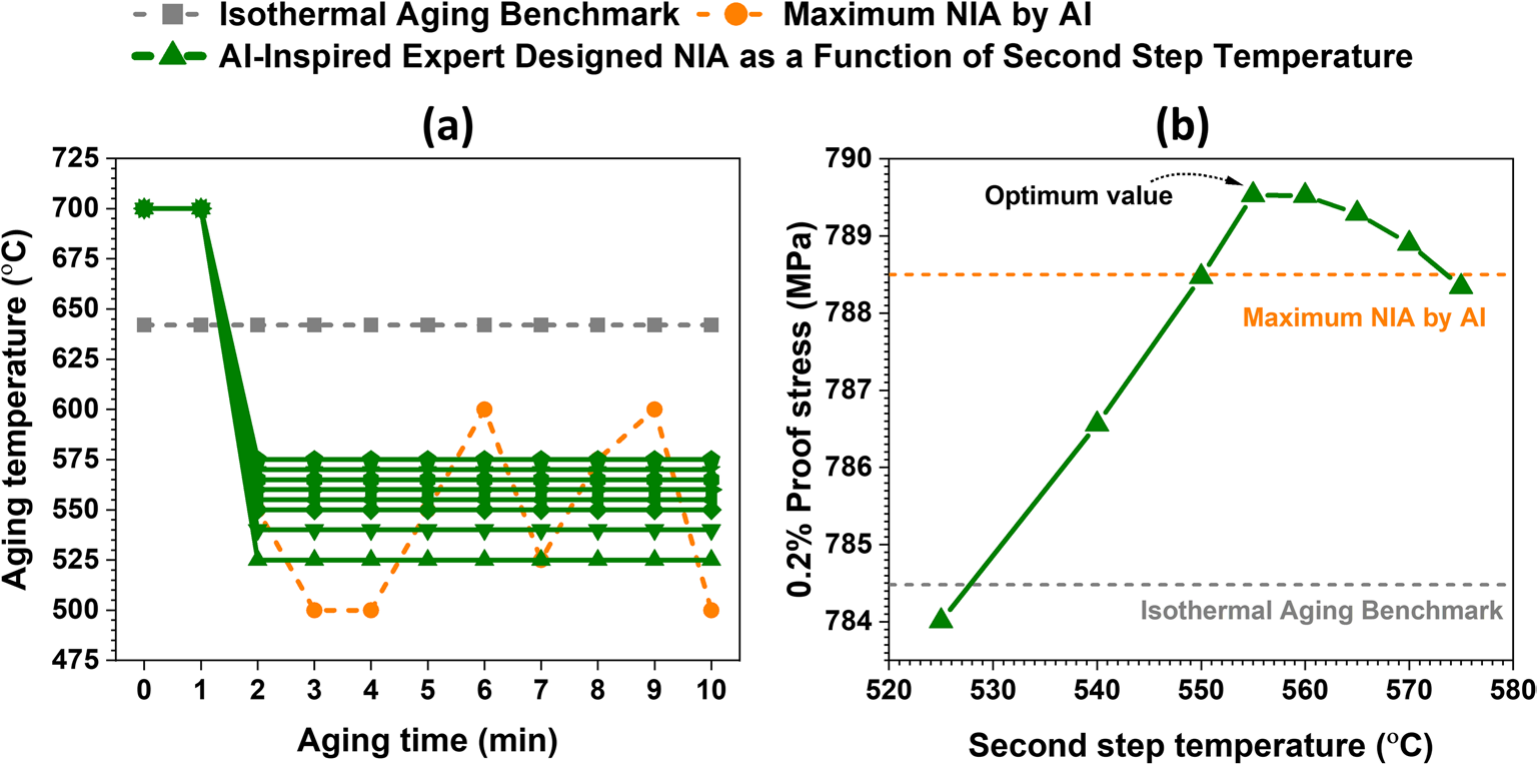
(**a**) Scheduling of isothermal aging benchmark, AI-assisted maximum 0.2% proof stress NIA and AI-inspired expert-designed NIA in this study and (**b**) variation of 0.2% proof stress as a function of second step temperature.

We here examined the optimal temperature for the second step in the range of 525–575 °C in order to attain the highest 0.2% proof stress with an efficient increase of the γ′ phase fraction, as shown in Fig. 12a. We found that 555 °C is the optimal second-step temperature yielding the maximum 0.2% proof stress, as shown by the black dotted arrow in Fig. 12b. Note that our newly proposed two-step aging with the optimal second-step temperature outperformed not only the isothermal aging benchmark (refer to grey dashed lines) but also the AI-found best NIA (refer to orange dotted lines). The results show the potential for collaborative creation between AI and experts in materials research. The comparison of 0.2% proof stress values is also tabulated in Table 3.

**Table 3:** Different aging routes and their corresponding 0.2% proof stresses (*ST* starting temperature).

Design	Aging scheduling detail	0.2% proof stress
Isothermal aging benchmark	642 °C–10 min	784.48 MPa
Maximum NIA by AI	ST 700 °C → 700 → 550 → 500 → 500 → 550 → 600 → 525 → 575 → 600 → 500 °C	788.50 MPa
Optimum AI-inspired expert-designed NIA	ST 700 °C, 700 °C–1 min, 700 °C → 555 °C (− 2.416 °C/s), 555 °C–8 min	789.53 MPa

**Figure 13:**
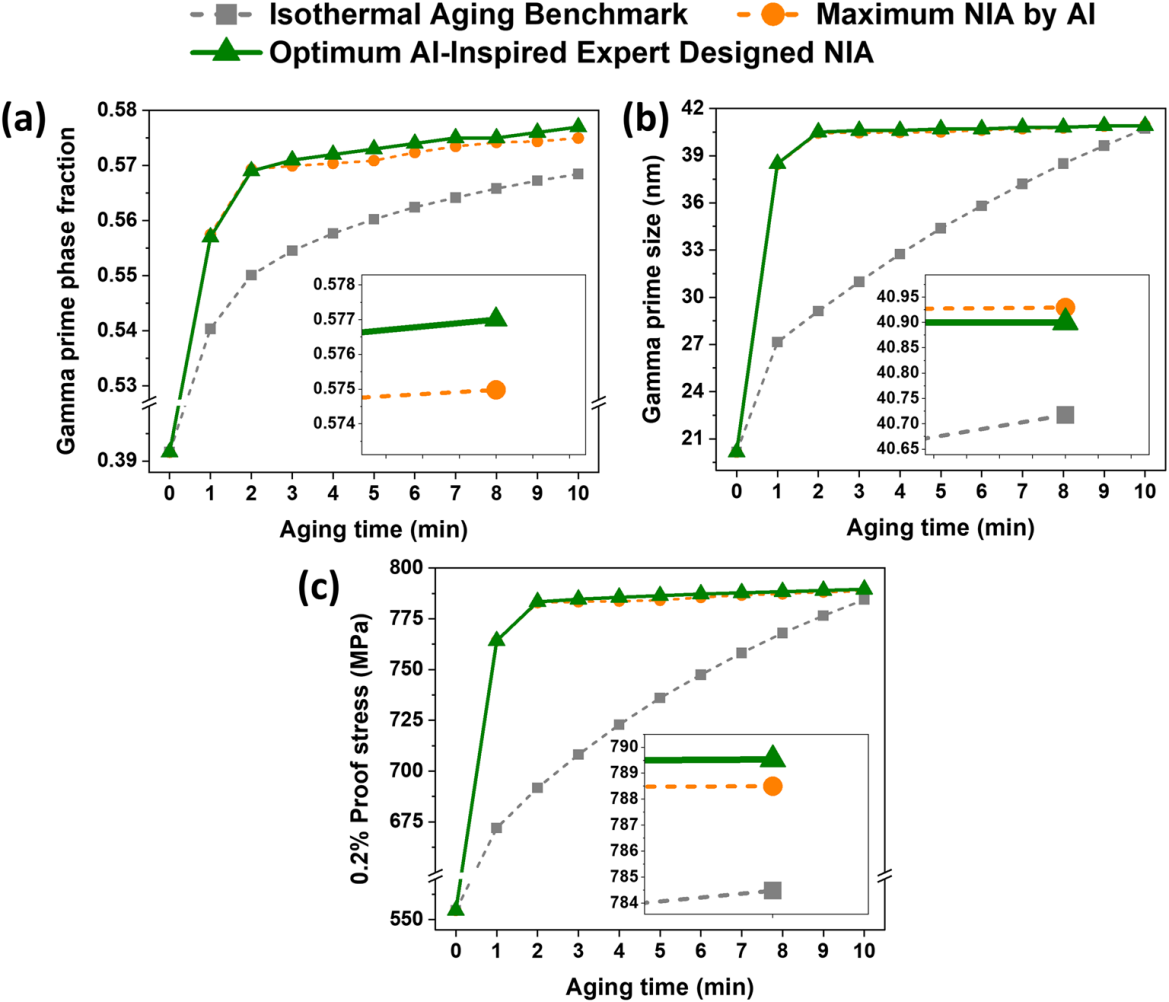
Comparison of optimum AI-Inspired expert-designed NIA with the isothermal aging benchmark and maximum NIA by AI in terms of (**a**) γ′ phase fraction, (**b**) γ′ size and (**c**) 0.2% proof stress as a function of aging time. Images of the tenth minute are zoomed-in in the insets (the reader is referred to the web version of this article for interpretation of the color references in this figure legend).

It is also important to compare the microstructural evolution characteristics such as γ′ phase fraction and size of the best-performed AI-Inspired expert-designed NIA with the isothermal aging benchmark and maximum NIA by AI (i.e., AI-assisted maximum 0.2% proof stress NIA). For instance, we aged the alloy at 700 °C for 1 min from the starting temperature of 700 °C (i.e., 1 min isothermal aging at 700 °C), then cooled it to 555 °C from 700 °C in 1 min (i.e., the cooling rate of $-$ 2.416 °C/s), and then maintained for 8 min at the same temperature (i.e., isothermal aging for 8 min at 555 °C). Figure 13a–c compare the AI-Inspired expert-designed NIA phase fraction, size, and 0.2% proof stress to those of the isothermal aging benchmark and AI max. NIA route (i.e., NIA 1 in Fig. 9), respectively.

The results demonstrate that the AI-Inspired expert-designed NIA has a slightly higher γ′ phase fraction than AI max. NIA, as shown in Fig. 13a. While the γ′ size of the AI-Inspired expert-designed NIA is less (i.e., near to critical size ~ 41 nm) than AI max. NIA, as shown in Fig. 13b. Hence, it results in improved 0.2% proof stress, as illustrated in Fig. 13c. The difference in 0.2% proof stress is clearly shown in the inset plot of Fig. 13c. One may relate this to the best combination of γ′ phase fraction and their size to achieve the optimum strength in the alloy. One hypothesis is that the early-stage high-temperature and later low-temperature heating may be able to bridge the best combination of γ′ precipitate size and γ′ phase fraction in these alloys. Therefore, early high-temperature heating helps in reaching the γ′ precipitate size near critical size (i.e., ~ 41 nm) and later lower temperature aging increases the γ′ phase fraction in these alloys.

We utilized Ni/Ni_3_Al two-phase alloy as an example in this study. The fundamental ideas, nevertheless, are rather general and should be applicable to various precipitate-hardening systems. This work may be the first step in the development of various heating scheduling methods employing machine learning, MCTS, in order to enhance strength and design NIA routes.

In conclusion, our study developed the pipeline to optimize the NIA schedules to maximize 0.2% proof stress at 725 °C for the Ni–Al (Ni-19.11 at. % Al) binary alloy with the γ–γ′ two-phase microstructure. The pipeline consisted of the computational workflow predicting the 0.2% proof stress in MInt and the MCTS, the AI algorithm finding the NIA routes efficiently. The search space was defined as follows. That is, the aging time was set to be 10 min, and the temperature was allowed to change in 25 °C steps in the range of 500–700 °C every minute. The number of possible different NIA schedules was huge, ${9}^{10}$ (i.e., 3,486,784,401). The MCTS found the 110 NIA schedules that outperformed the isothermal aging benchmark in terms of 0.2% proof stress. The detailed analysis of the AI-found top 5 NIA schedules revealed that they commonly consisted of two stages, as follows: early high-temperature aging for a shorter time to rapidly increase the γ′ precipitate size to the near-critical size ~ 41 nm and the subsequent lower temperature aging to increase the γ′ phase fraction by keeping the γ′ under the critical size. Based on the essence of the AI-found NIA revealed by the analysis, we proposed a new concept of two-step aging consisting of 1-min isothermal aging at 700 °C, cooling down to a lower temperature, and 8-min isothermal aging at a lower temperature. We found the optimum lower temperature for the second step to be 555 °C for the considered alloy and then confirmed that this AI-Inspired NIA outperforms the AI-found best one. The design methodology using the AI-found solutions as a source of inspiration and the newly proposed two-step aging concept based on the methodology should be effective for Ni-base superalloys with a similar γ–γ′ two-phase microstructure.

### Supplementary Information

Supplementary Information.

## Data Availability

The datasets used and/or analysed during the current study available from the corresponding author on reasonable request.
