# Supplementary material for: Artificial intelligence inspired design of non-isothermal aging for γ–γ′ two-phase, Ni–Al alloys
Source: Sci Rep. 2023 Aug 4;13:12660. doi: 10.1038/s41598-023-39589-2 (PMC10403502; doi:10.1038/s41598-023-39589-2)
Supplement: Supplementary file 1 — Supplementary Information. [file 41598_2023_39589_MOESM1_ESM.docx]

**Supplementary Material for:**

**Artificial Intelligence Inspired Design of Non-Isothermal Aging for**

**γ -** **γ' Two-phase Ni-based Alloys**

Vickey Nandal^1^*, Sae Dieb^1^, Dmitry S. Bulgarevich^1^, Toshio Osada^1^, Toshiyuki Koyama^2^, Satoshi Minamoto^1^, Masahiko Demura^1^*

*^1^National Institute for Materials Science (NIMS), Tsukuba, Japan*

*^2^Department of Materials Design Innovation Engineering, Nagoya University, Nagoya, Japan*

Corresponding Authors: [nandal.vickey@nims.go.jp](mailto:nandal.vickey@nims.go.jp)*, [demura.masahiko@nims.go.jp](mailto:demura.masahiko@nims.go.jp)*

Fig. S1 shows the quantitative analysis of the microstructure evolution of the distribution of γ' (gamma prime)
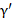
particles under non-isothermal aging (NIA) conditions in Ni-19.11 at. % Al alloy with the help of the MInt system. The results in terms of phase fraction of
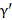
 γ' precipitates, their size (i.e., the mean diameter of a circle of equal projection area for γ'-precipitates) and 0.2% proof stress are displayed in Fig. S1. It indicates that the phase fraction of
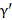
 γ' particles increases when the temperature increases rapidly (Fig. S1a), and their average size also increases with aging time (Fig. S1b). However, after a while, the particle becomes unstable at higher temperatures, which results in a drastic increase in γ' size or coarsening (i.e., over-aging) that is detrimental to mechanical properties, as demonstrated in Fig. S1c. For example, the increase in γ' phase fraction within a minute is evidently very sluggish up to 600 °C and then increases rapidly after that, as shown in Fig. S1a. It is interesting to note that after achieving the isothermal aging benchmark at 642 °C, the size of the γ' particle increases significantly (see Fig. S1b), and when the size crosses ~ 41 nm mark, the magnitude of 0.2% proof stress drops, as shown in Fig. S1c. For instance, in the case of 650 °C, the 0.2% proof stress began to decrease after 8 minutes of aging once the γ' size reached at 41.5 nm (indicated by the light brown color arrow in Fig. S1b). The γ' phase fraction stays approximately unchanged after reaching the highest 0.2% proof stress (i.e., peak-aging state). As a result, the strength is effectively controlled by the increment of γ' size over the critical size. A similar pattern was observed at the aging temperatures of 675 °C (indicated by red color arrow) and 700 °C (indicated by black color arrow). Hence, in this work, the critical size of the γ' precipitate is assumed to be around 41 nm.


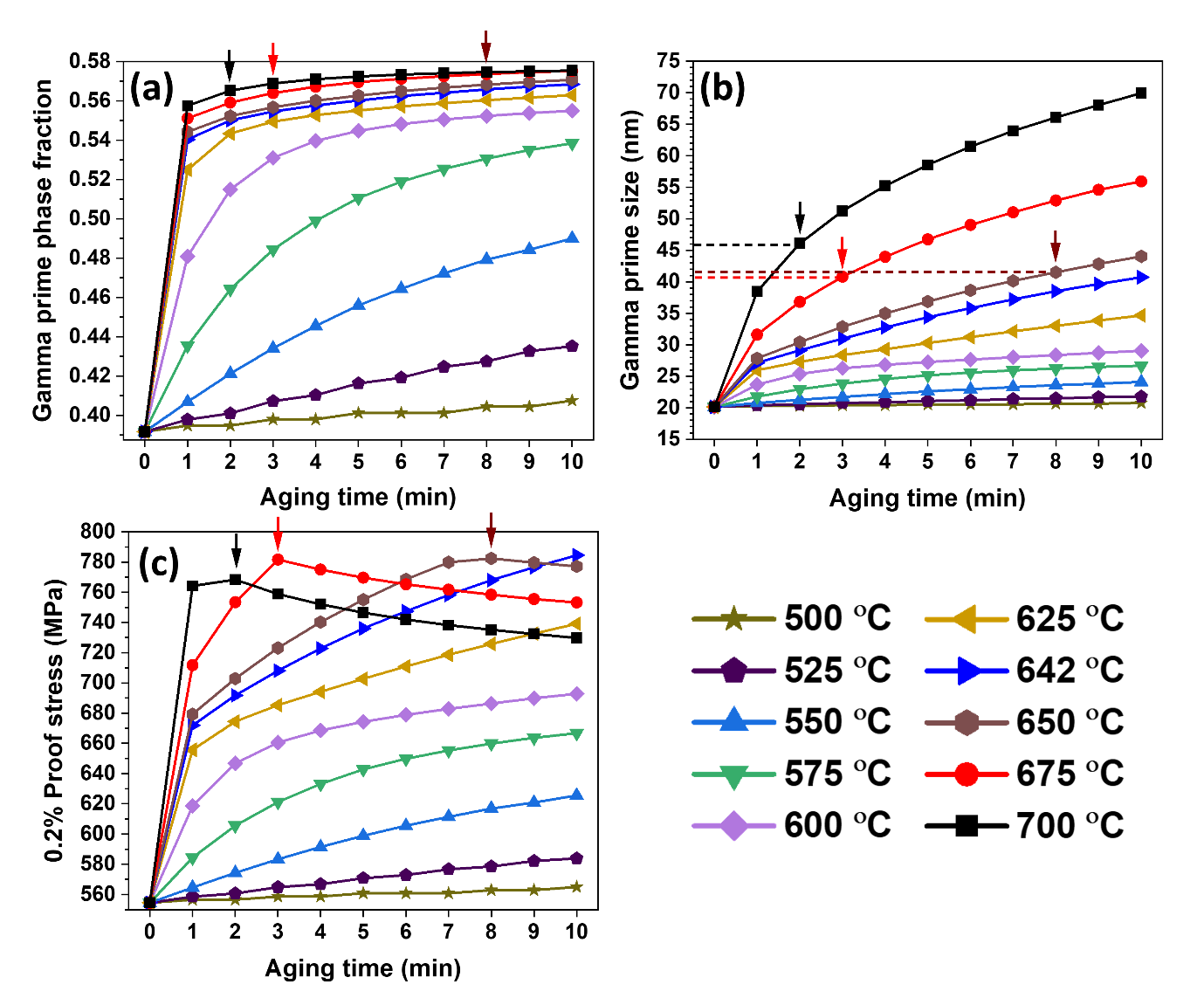


**Figure S1:** Plots of modeled precipitation evolution as a function of aging time in terms of (a) gamma prime phase fraction, (b) size and (c) 0.2% proof stress.

The modeled results are also plotted as 2D contour plots, as shown in Figs. S2a – c. The contour plots indicating the measured γ' phase fraction, size and 0.2% proof stress for Ni/Ni_3_Al two-phase alloy are shown in Fig. S2a, b and c, respectively. It is apparent that the increase in aging temperature (i.e., above 625 °C) accelerates the γ' phase fraction and size (i.e., critical diameter) and improves the strengthening response of the alloy. It is clear that temperatures below 625 °C have little effect on the phase fraction and size, even less than the critical size, which directly affects the 0.2% proof stress (refer to Fig. S2c). Star symbols indicate the benchmark condition for isothermal aging in the contour plots.


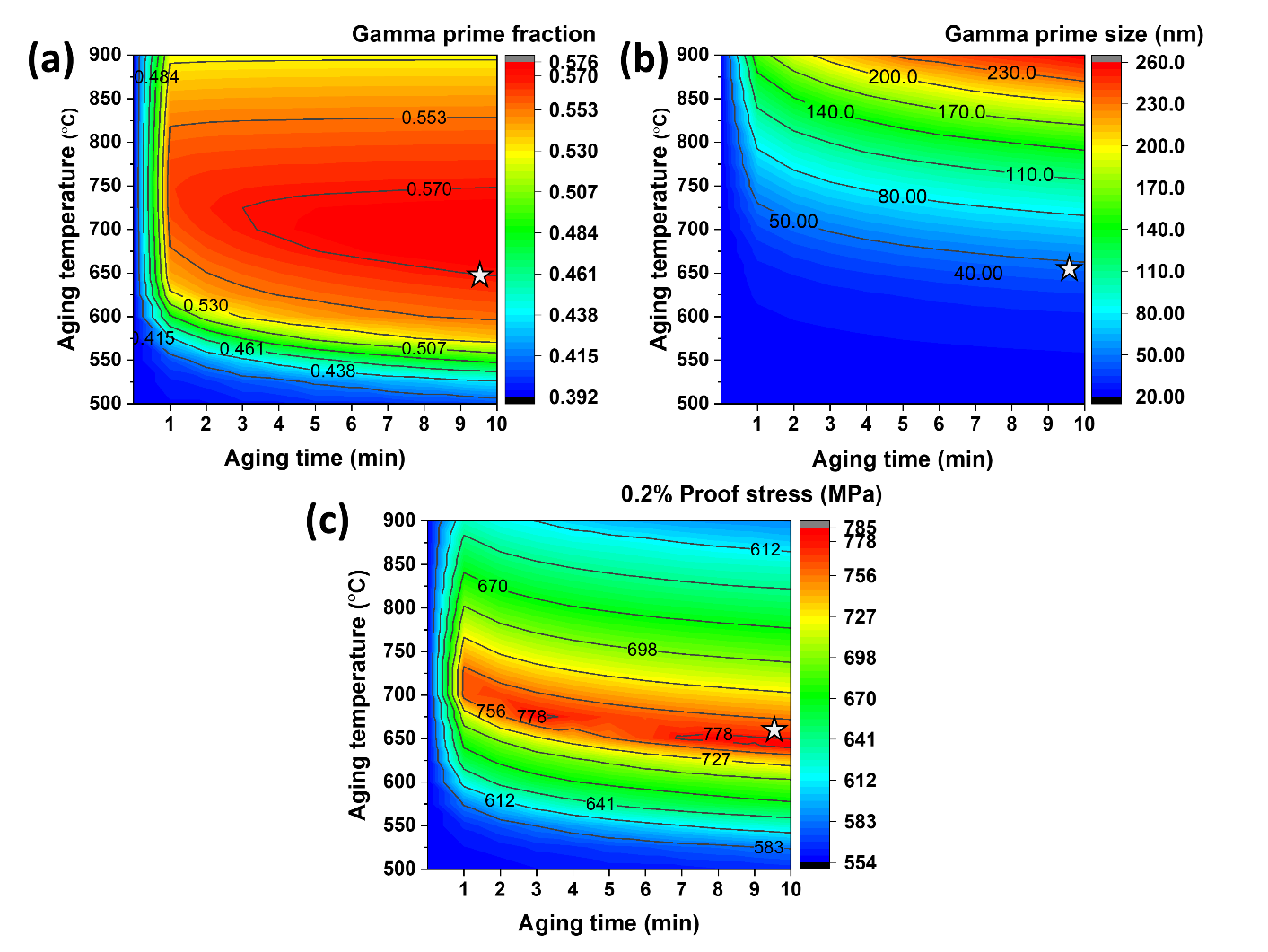


**Figure S2:** The 2D contour plots for the (a) γ' phase fraction, (b) size and (0.2% proof stress as a function of aging temperature. The star symbol indicates the benchmark condition for isothermal aging. (The reader is directed to this article's web version to clarify the color references in this figure legend).
